# Visualizing the hydrodynamics in sieve-based lateral displacement systems

**DOI:** 10.1038/s41598-018-31104-2

**Published:** 2018-08-27

**Authors:** J. P. Dijkshoorn, J. C. de Valença, R. M. Wagterveld, R. M. Boom, M. A. I. Schutyser

**Affiliations:** 1 Laboratory of Food Process Engineering, Wageningen University, Bornse Weilanden 9, 6708WG Wageningen, The Netherlands; 2 Wetsus, European Centre of Excellence for Sustainable Water Technology, Oostergoweg 9, 8911MA Leeuwarden, The Netherlands

## Abstract

Deterministic lateral displacement (DLD) systems structure suspension flow in so called flow lanes. The width of these flow lanes is crucial for separation of particles and determines whether particles with certain size are displaced or not. In previous research, separation was observed in simplified DLD systems that did not meet the established DLD geometric design criteria, by adjusting the outflow conditions. We here investigated why these simplified DLD systems are able to displace particles, by experimentally investigating the hydrodynamics in the device. Flow lanes were visualized and the local flow velocities were measured using µPIV and compared with 2D fluid dynamics simulations. The size of the flow lanes strongly correlates with the *local* flow velocity (V_y_ and V_x_), which depends on the hydrodynamics. Therefore, the geometric design criteria of DLD devices is in fact just one method to control the local hydrodynamics, which may also be influenced by other means. These findings give a new perspective on the separation principle, which makes the technique more flexible and easier to translate to industrial scale.

## Introduction

Separating neutrally buoyant suspensions of micron-sized particles (1–10 µm) is not a trivial operation. An effective technique to separate large volumes of these particle suspensions is microfiltration, but even this technique suffers from drawbacks like concentration polarization, cake layer formation, pore blocking and internal pore fouling^1–3^. Therefore, alternative separation techniques have been proposed that make use of microfluidic separation principles^4–7^. Many of these microfluidic separation principles may show potential, but they need significant redesign to enable upscaling to larger volumes. Adaptation of the design may render these systems more suited for processing large volumes, but this makes it essential to characterise the hydrodynamics of the redesigned system. This can be done by combining visualization methodologies, such as Particle Image Velocimetry (PIV) with Computational Fluid Dynamics (CFD)^8–10^.

**Figure 1:**
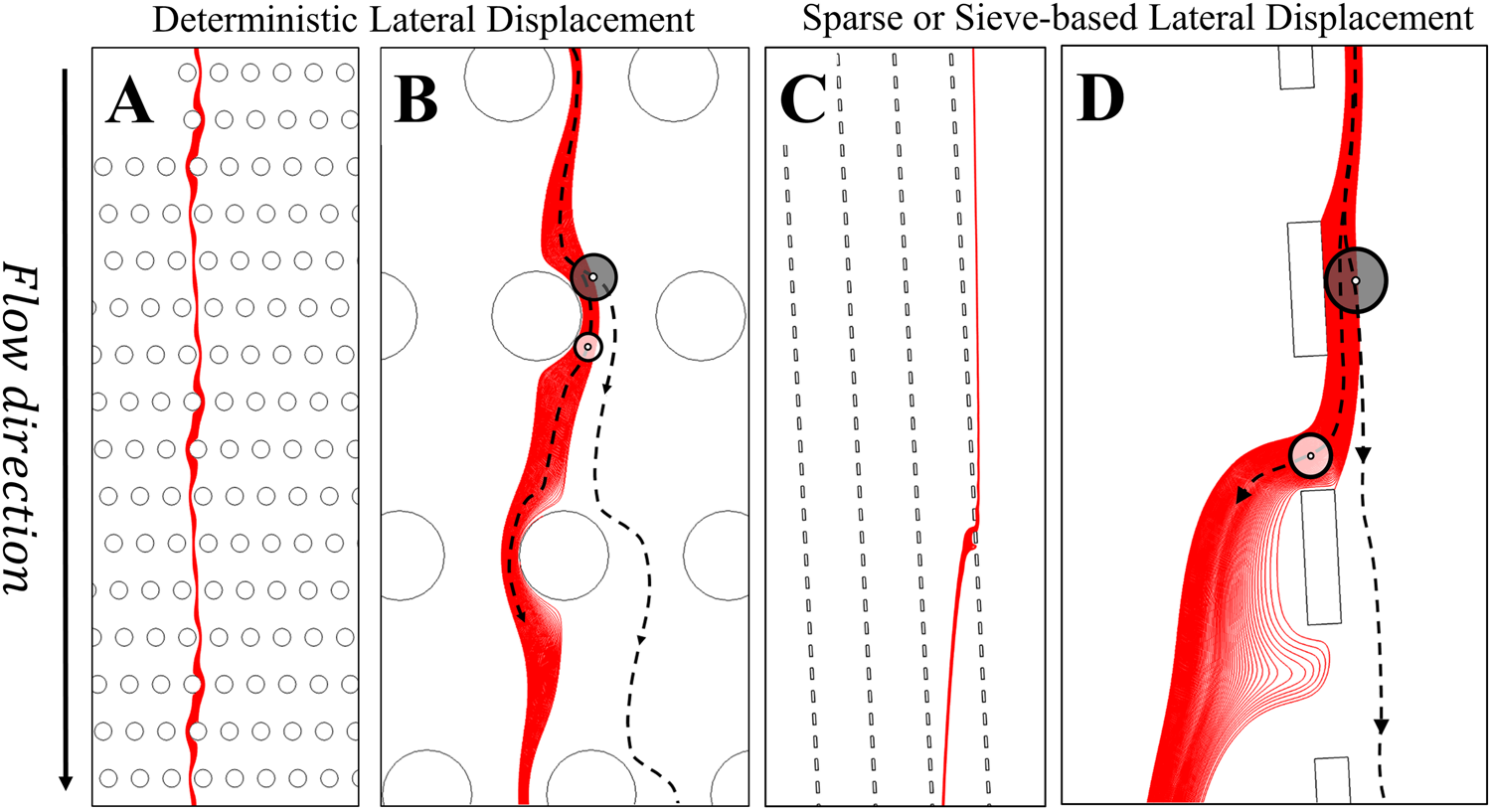
Flow lanes (red) in a part of the DLD array (**A**,**B**) and in the (bottom) part of the sieve-based lateral displacement (SLD) system (**C**,**D**). (**A**,**C**) shows the flow lanes in a larger part of the device. (**B**,**D**) Shows a close up of the same flow lanes with the trajectories of two particles. Particles larger than twice the critical flow lane diameter (grey) are displaced and follow the angle of the obstacle column. Particles smaller than this critical diameter (white) follow the flow lane and flow through the gap.

Deterministic lateral displacement (DLD) systems are promising for (large-scale) suspension separation, because it can separate particles that are smaller than the gaps or pores in the system, limiting the risk of blockage. As a consequence, the systems are expected to be less sensitive to fouling than a microfiltration system, while the required pressure drop is lower and the design of the peripheral system is simpler, lowering the capital and operational costs. However, DLD devices in their microfluidic design are difficult to scale up and not yet suitable for processing large volumes. Asymmetric DLD systems such as a sieve-based lateral displacement (SLD) system are more promising for scale-up (Fig. 1A vs. Fig. 1C) because they (1) are less prone to foul, (2) have an even lower pressure drop, and (3) are easier to manufacture and can be constructed with existing microsieves (three-dimensional geometry)^1,6,11–14^.

Separation in DLD (planar geometry) and SLD systems (non-planar geometry) relies on particle-obstacle interactions that laterally displace particles in the fluid from their streamlines, out of the critical flow lane^15^. A flow lane (red lane in Fig. 1) is defined as the set of streamlines that passes between two subsequent (longitudinally adjacent) obstacles. Each gap possesses a flow lane. When a particle is larger than twice the width of the flow lane, at the location when it is about to flow between two obstacles, particle-structure interaction will laterally displace this particle (grey) from its initial flow lane into the next (Fig. 1B,D and supplementary video). If the particle radius is smaller than the flow lane width, this particle (white) may still be displaced but not sufficient to cross over to the next flow lane and therefore will stay in its initial flow lane (Fig. 1B,D and supplementary video). This means that the critical particle diameter is controlled by the width of the flow lanes, which makes precise control of the flow essential. In most previous published studies the flow is controlled by (periodic) geometric design constraints (e.g., angle, gap sizes, obstacle size/shape) by adjusting inlet velocity^16^ and/or outflow conditions^12^. In systems meant for large-scale separations, one wishes to minimise the presence of obstacles, while the system should not be too dependent on the precise local velocity and outflow condition (e.g. a SLD system). In addition, the influence of particle-particle interactions and the influence of particles on the flow are neglected and we assume that separation only depends on the flow lane width. This is not valid for separations with a significant volume fraction of particles to separate.

The influence of the geometric design criteria of DLD arrays on the critical particle diameter has been thoroughly investigated. The hydrodynamics in such systems have, however, not received much attention, although it is known to influence the size of the flow lanes. Improved control of the hydrodynamics for instance by adjusting the outflow conditions can provide more design freedom and make production easier and cheaper^12,14^. Specifically, we address the hydrodynamics in a sieve-based lateral displacement (SLD) system.

The objective of the study reported here is to characterize the hydrodynamics and its influence on flow lane size. The flow lanes were experimentally visualized and the local velocities were measured in a SLD device with different inlet flow rates. These measurements were compared with 2D numerical simulations. Subsequently, these simulations were used to correlate the size of the flow lanes with the local velocity.

## Results and Discussion

We first visualized the flow lanes (2.1) with a high speed camera. Second, we used micro-particle image velocimetry (µPIV) to measure the local flow velocities and compare these with the 2D simulations (2.2). These 2D simulations were then used to correlate the width of the flow lanes with the flow velocity (2.3).

### Visualization of the flow lane in a sieve-based lateral displacement device

**Figure 2:**
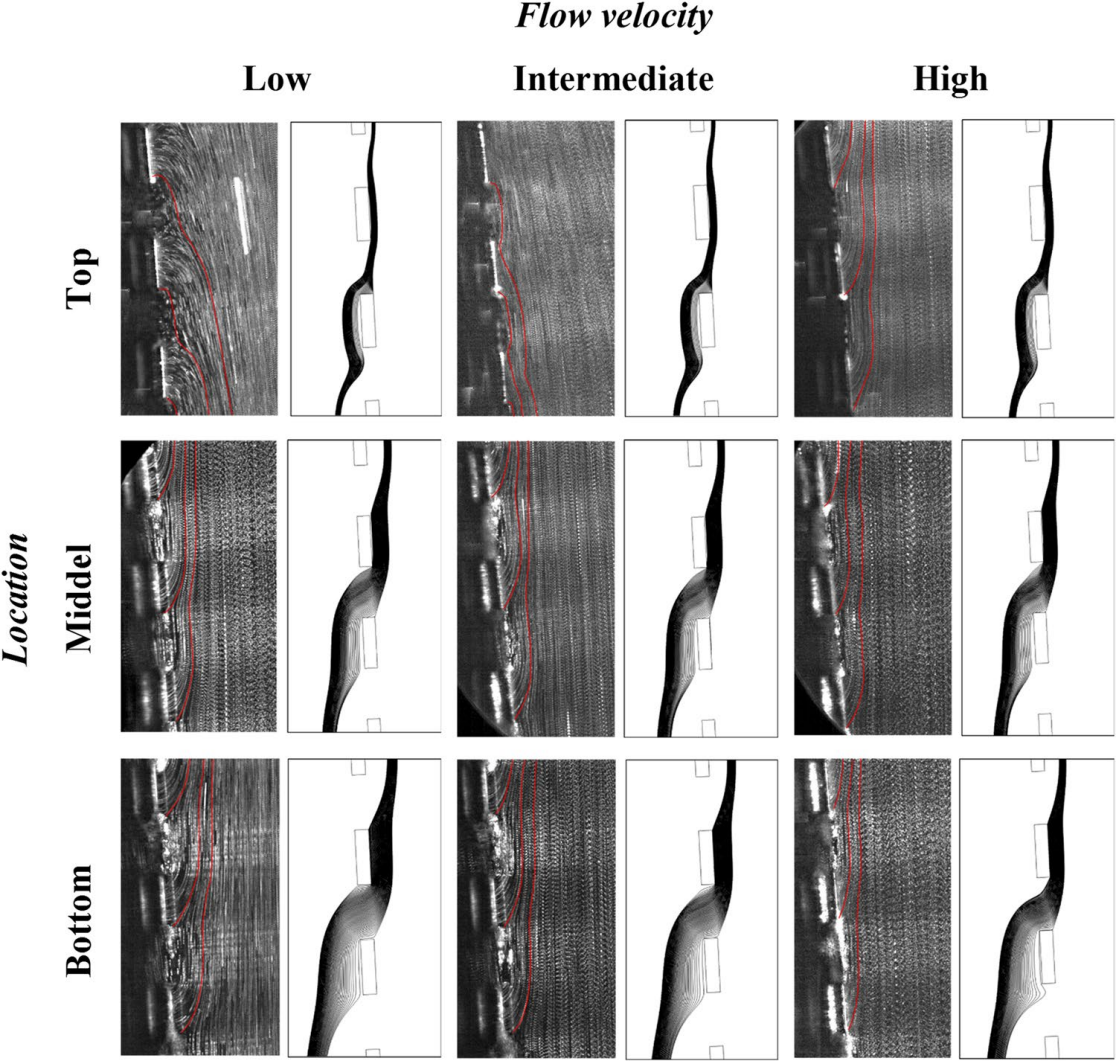
Experimental and numerical visualization of the flow lanes in a sieve-based lateral displacement device. From left to right, the inlet velocities increases (between 0.1 and 0.12 m/s), from top to bottom are three locations along the right sieve (Fig. 3). The red lines are introduced to distinguish between the pathlines that will flow into the pore and that will go straight, the pathlines clustered together that flow into a pore are considered a flow lane. The flow lanes at the top are not steady because the flow in this region was turbulent. The 2D numerical simulations assume laminar inflow.

Critical flow lanes were experimentally visualized in a sieve-based lateral displacement device on three locations and with three inlet velocities (Fig. 2). The flow lane width was visualized using the trajectories of individual tracer particles and the most outward pathline that enters or exits this gap is outlined by a red line. These flow lanes were then compared with the flow lanes obtained from the 2D fluid model, which did not contain particles.

Near the entrance of the system, the experimental flow pattern deviated from the 2D fluid simulations. This was caused by the inlet configuration which induced a jet and resulted in some turbulence at the entrance region of the experimental setup. The turbulence flow resulted in flow instability over time, and the suspension intermittently flowed into and out of the pores at the top of the system. These variations made it impossible to measure stable flow lanes. For the 2D simulations, the entrance flow was assumed to be laminar and stable over time, which allowed us to derive the flow lane width. These simulated flow lane widths were smaller at lower inlet velocity, and gradually grew larger with increasing inlet velocity. Furthermore, the simulated flow lanes in the top section were narrower compared to the flow lanes in the bottom section. Though, the experimental results and simulations at the top of the system are not alike, separation has yet to take place. The bottom region is more important because at this location significant particle displacement should have taken place. Because the experimental observed flow lanes and simulated flow lanes are similar at the bottom of the system we used these simulations for further analysis.

Flow lanes were measured and visualized in the middle and bottom sections of the device, because here the flow had stabilized and became independent from the inlet disturbances (Fig. 2 and Table 1). In general, the width of the flow lanes varies depending on the location and the inlet velocity. A higher inlet velocity reduced the width of the flow lanes. The widest observed flow lane was 106 µm, which means that the cut-off particle size is exactly the pore size; particles in this flow lane that have a diameter smaller than that of a pore (200 µm) will always flow into this pore and larger particles will be excluded. Wide flow lanes are, therefore, not desired.

**Table 1:** Size of the flow lanes are shown in Fig. 2.

	Low velocity		Intermediate velocity		High velocity	
	*PIV*	*Simulations*	*PIV*	*Simulations*	*PIV*	*Simulations*
Top	NA	25 µm	NA	27 µm	NA	30 µm
Middle	71 ± 7 µm	64 µm	64 ± 5 µm	61 µm	63 ± 5 µm	56 µm
Bottom	106 ± 8 µm	77 µm	81 ± 6 µm	65 µm	61 ± 5 µm	55 µm

Experimental obtained flow lane sizes (mean ± standard deviation) are measured for 3 pores in 15 stacks of 100 frames (45 measurements per flow lane) and obtained using 2D numerical simulations in COMSOL.

The flow lane width is known to be influenced by the velocity profile^14,17,18^; an asymmetric velocity profile is beneficial for separation because it reduces the width of the flow lanes^19^. This, however, is only true if the flow lanes through all gaps carry equal flux, which is assumed in (periodic) DLD systems^15,17^. This assumption is not valid in case of sparse or sieve-based lateral displacement systems and is expected also not to hold for systems with anisotropic permeability^14,20–22^. A highly asymmetric velocity profile does not by definition reduce the width of the flow lanes. We hypothesize that there is a balance between the vertical velocity component (V_y_) due to the inlet flow, and the horizontal velocity component (V_x_) of the fluid, due to the flow into the gaps. When V_y_ and V_x_ are balanced over the entire length of a system, the flow lanes will have the same width throughout the system. And if V_y_ grows with respect to V_x_ the flow lane should become narrower and vice versa. To confirm this hypothesis, the velocity field was measured at the same locations where the flow lanes were visualized. The V_y_ and V_x_ components were analysed from the acquired data. The velocity profiles obtained with these µPIV measurements are compared with the velocity profiles calculated with the 2D numerical simulations.

### Velocity profile obtained with µPIV and 2D numerical simulations

**Figure 3:**
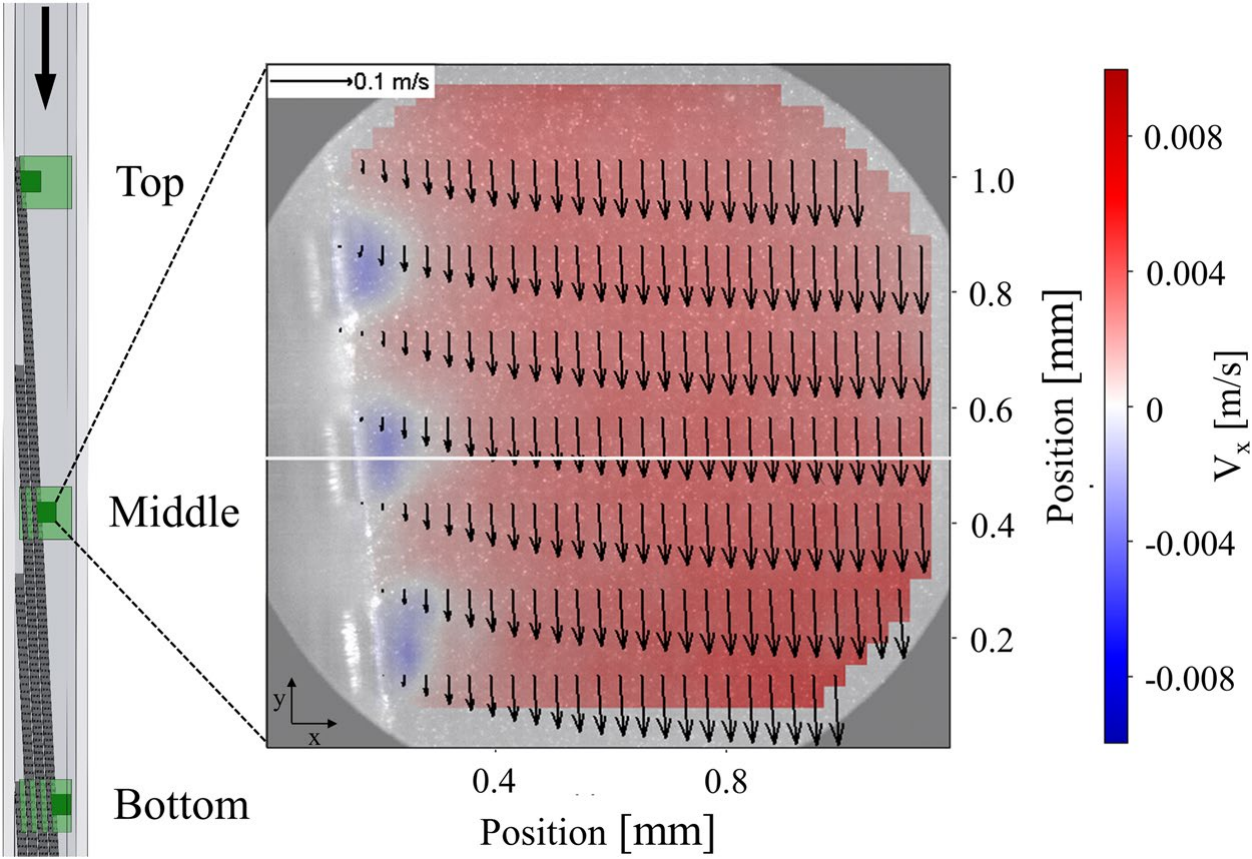
Schematic overview of the experimental setup and the recording locations: top, middle and bottom for both the entire channel (light green square) and the pores (dark green square). The vector field shown (every fourth vector row) is taken near the pores and is recorded at in the middle of the device, where the colour indicates the V_x_ (blue is left and red is right). The white line gives an example of a measurement location for the average velocity profile (Fig. 4D,F). The vector field shows the absolute velocity and the colour indicates only V_x_.

The flow velocity profile was measured for three different inlet velocities and at three locations in the device (Fig. 3). Two sets of recordings were made for each location: a recording of the entire channel width (light green squares) and a detailed recording of the largest sieve (dark green squares). The recordings were translated into an average vector field by using PIV software (Fig. 3). The vectors indicate the size and direction of the composite velocity, the background colour indicates the magnitude of the transverse velocity component V_x_.

These average vector fields were used to collect V_y_ and V_x_ profiles along a line that passes through the centre of a pore, for example the white horizontal line in Fig. 3. V_y_ was acquired from the entire channel (light green square), which also allowed us to determine the flow velocity behind the sieves. For an accurate estimation of V_x_ near a pore gap it was necessary to zoom in onto the sieve (dark green square), which allowed the observation of tracer particles flowing towards the pores.

**Figure 4:**
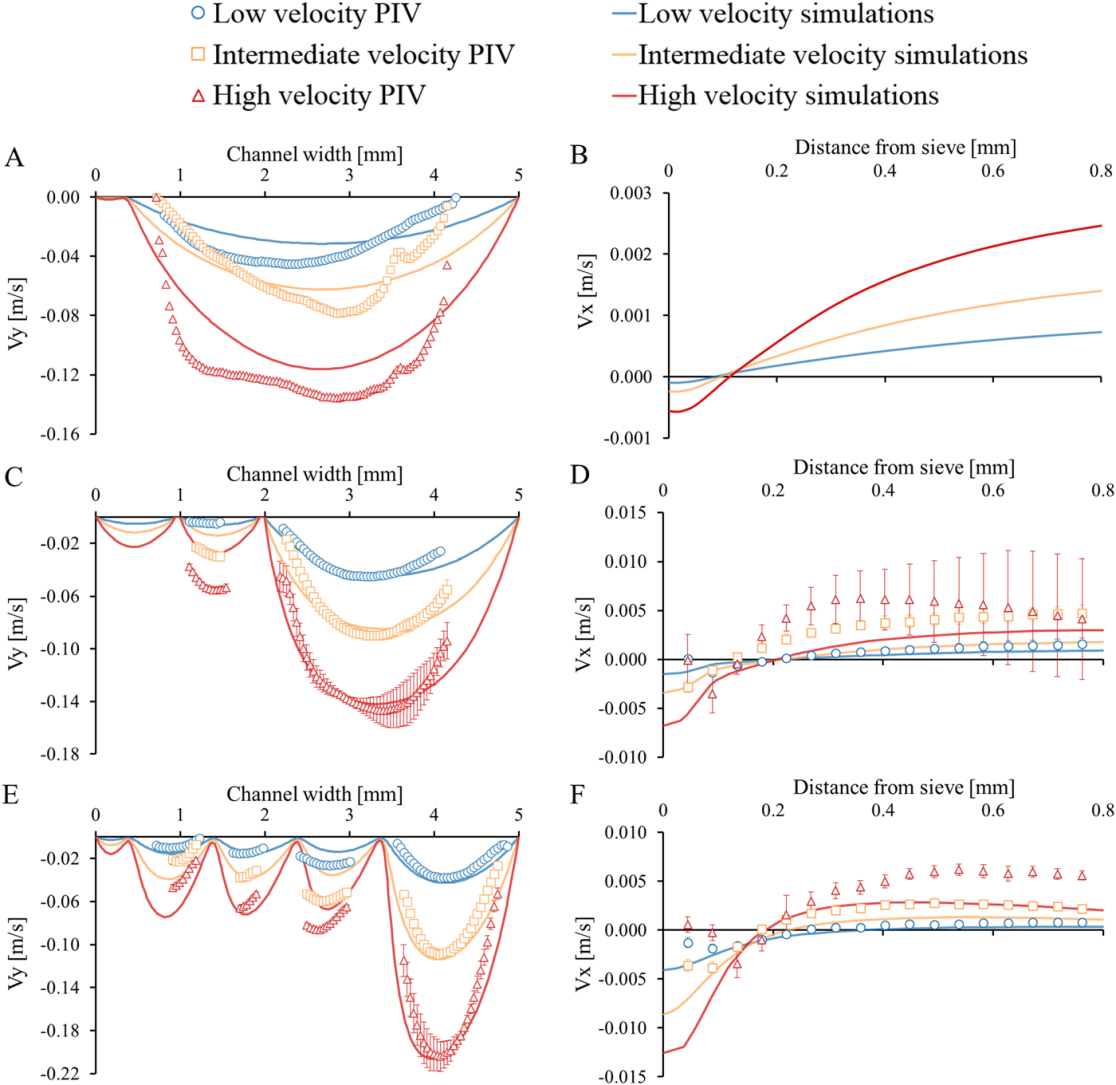
Velocity profiles (V_y_ in ACE and V_x_ in BDF) at three locations in the device (AB at the top, CD in the middle and EF at the bottom) for three average inlet velocities (low is 0.02 m/s, intermediate is 0.04 m/s and high is 0.075 m/s). The experimental data shows the mean velocity and SD and the y-axes are not equal. The data points of (**A**) are an average V_y_ of 1500 images and the SD is not shown. The measured V_x_ is not shown in (**B**) because the flow was not stable.

The measured and simulated velocity profiles are shown for the three locations and three inlet velocities (Fig. 4). The velocity profiles for both V_y_ and V_x_ (negative direction) increase towards the bottom of the device. The actual inlet flow velocities during the experiments were estimated with the 2D simulations because the flow rate of the pump was influenced by the pressure drop at higher flow rates. The low inlet velocity was ~0.02 m/s (Re ~100), the intermediate inlet velocity was ~0.04 m/s (Re ~200) and the high inlet velocity was ~0.075 m/s (Re ~375). Slight differences in channel width can be observed between the experimental system and the 2D simulations, which may be caused by irregularities in the construction (e.g. sieves and channel surface) or by inaccuracies in the imaging (e.g. camera position and/or lighting).

The velocities measured in the top section of the device are presented in Fig. 4A. At this location the flow was turbulent due to a jet created at the entrance of the device. Therefore, the results are averaged over ~1500 images, and the standard deviation (SD) is not shown ( ± 0.04 m/s). We do not show the experimental V_x_ profile in Fig. 4B because of strong flow instabilities. Only a small amount of fluid flows through the pores (negative velocity), while most of the fluid flows away from the sieves (positive velocity).

The velocities measured in the middle section are shown in Fig. 4C,D. At this location the flow had become laminar, and V_y_ could be measured both in front and behind the sieve (Fig. 4C). The experimental V_x_ component was negative near the sieves indicating that the fluid indeed flowed towards the sieves and through the pores. While this is qualitatively in line with the simulations, the magnitudes of the velocities were somewhat different (Fig. 4D). Towards the channel centre, the direction of V_x_ reverses and the fluid started to flow away from the sieves.

Figure 4E presents values for the V_y_ component between multiple sieves in the bottom section of the device for both simulations and experiments. The V_x_ component is shown in Fig. 4F. The agreement between experiment and simulation is acceptable. The flow could not be visualized inside the pores and consequently, the experimental measurements cannot be used to find a correlation between the size of the flow lanes and the flow velocity (V_y_ and V_x_) inside the gap. Therefore, the 2D simulations are used instead for this.

### The balance between V_y_ and V_x_, and its influence on the flow lanes

**Figure 5:**
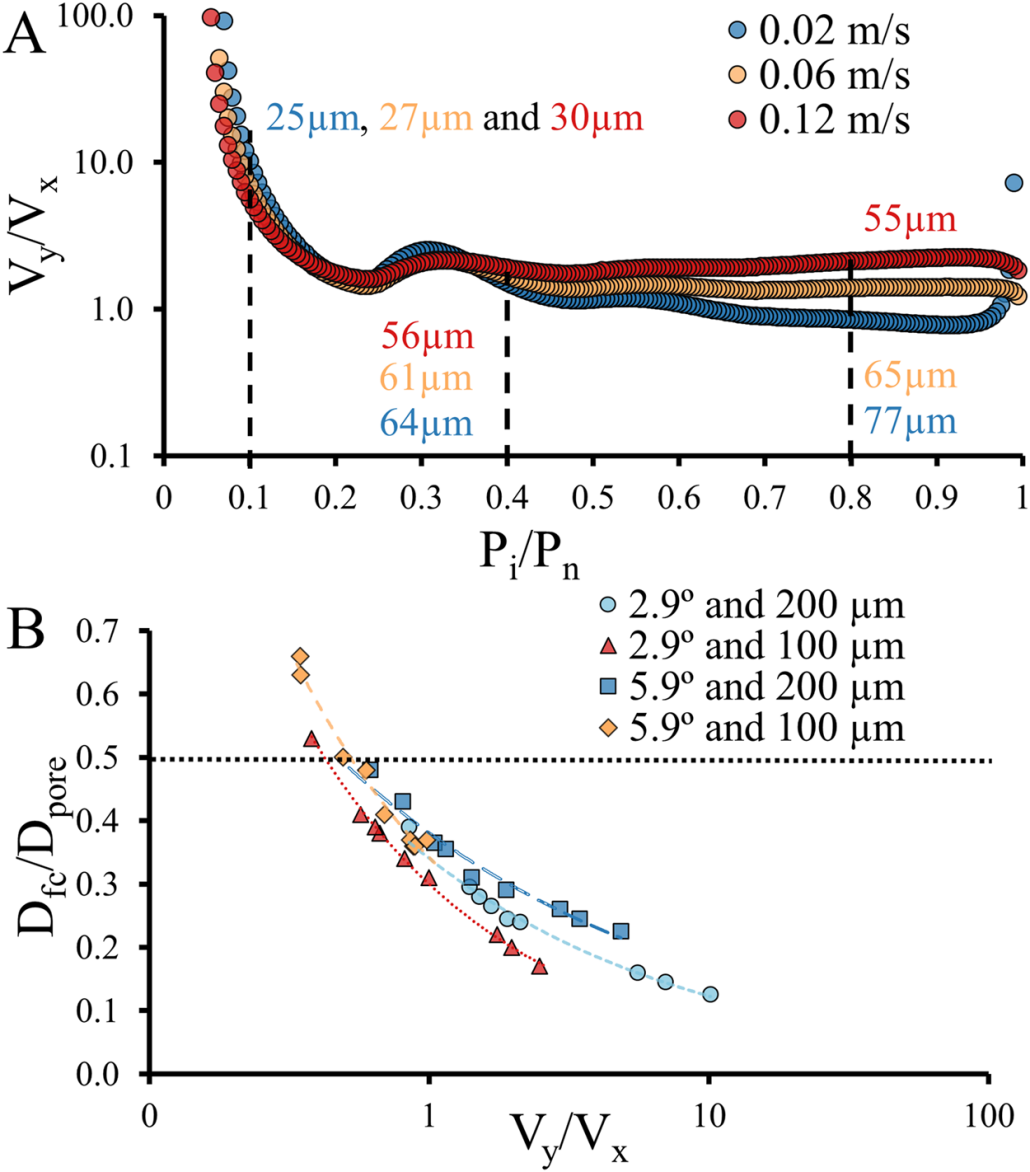
(**A**) For this system the ratio of velocity components (V_y_/V_x_) in each pore was plotted on a logarithmic scale for the three inlet velocities. The pores were normalized by the total number of pores. The size of the numerically simulated flow lanes (Fig. 2 and Table 1) were indicated at top (0.1), middle (0.4) and bottom (0.8). A model was used with the same geometry as our experimental system, where the sieves were placed at an angle of 2.9° and had pores of 200 µm. (**B**) The size of nine flow lanes (D_fc_) relative to the pore size (D_pore_) were plotted against the V_y_/V_x_ ratio, where each marker represents one location and one inlet velocity. This was done for four systems with varying angle and pore size. A higher V_y_/V_x_ ratio decreased the flow lane width relative to the size of the pores. A minimum V_y_/V_x_ is required to displace particles because if D_fc_ ≥ 0.5*D_pore_, particles will not be displaced but filtered instead (dotted line).

A two dimensional model was made of our experimental setup, which was used to correlate the velocity components (V_y_ and V_x_) with the flow lanes (supplementary information). These flow velocity components were integrated over a cutline in the entrance region of each pore, the ratio of these components (V_y_/V_x_) is shown in Fig. 5. This velocity ratio is presented as function of the number of pores relative to the total number of pores along the sieve (P_i_/P_n_), where P_1_/P_n_ ≈ 0 indicates the first pore at the top and P_n_/P_n_ = 1 the last pore in the sieve at the bottom. This number therefore represents a spatial coordinate along the sieve.

Due to the design of the system, the V_x_ component at the top was near zero and therefore, the ratio of the velocity components was very high between P_i_/P_n_ = 0–0.1. The V_x_ component quickly increased in the downstream direction but V_y_ increased as well (Fig. 4A,C,E). The velocity ratio (V_y_/V_x_) eventually stabilized around a value of 2 (Fig. 5A), but this ratio was not completely stable and fluctuated somewhat near P_i_/P_n_ = 0.3, which corresponds to the start of a new parallel sieve (see also Fig. 3). This parallel sieve locally decreases the V_x_ and thus results in a small increase of V_y_/V_x_. This effect is apparent at 0.3 and is repeated to a smaller extent around P_i_/P_n_ = 0.55, where a third sieve starts. The final ratio is slightly different for the three inlet velocities, which means that depending on the inlet velocity, V_y_ changes relative to V_x_ (Fig. 5A).

Correlation of the width of the flow lanes (Table 1) with the corresponding velocity ratios, shows that a lower V_y_/V_x_ resulted in wider flow lanes, and thus separation of larger particles. This effect was visualized for three locations and three inlet velocities in four different system designs (Fig. 5B). Figure 5B illustrates the same trend observed in Fig. 5A, where a higher V_y_/V_x_ results in smaller flow lanes. The sizes of the flow lanes were normalized with the size of the pores (D_fc_/ D_pore_), which are shown as function of the V_y_/V_x_ ratios. The circular symbols in Fig. 5B represent the same nine flow lanes (three velocities and three locations) as described in Fig. 5A. The influence of V_y_/V_x_ on D_fc_/D_pore_ was investigated for three additional designs with varying angles (5.9° and 2.9°) and pore size (100 µm and 200 µm) to compare the effect of the geometry. Similarly shaped curves were obtained, with slightly different values per geometry. This is expected as the flow does not always behave linearly to changes in the geometry. Therefore, there is no unified description of V_y_/V_x_ for a specific flow lane size in devices with a different design.

Using the results in Fig. 5B, the performance of the four designs can be discussed. Displacement of the smallest possible particles with the largest possible pores requires flow lanes that are much smaller than the pores and thus, D_fc_/D_pore_ should be as low as possible. In contrast, with D_fc_/D_pore_ ≥ 0.5, the width of the flow lane is equal or larger than half the size of a pore and particles are no longer displaced but will either flow through the pore or are physically blocked instead. This limit is indicated by the black dotted line in Fig. 5B. A low D_fc_/D_pore_ requires a high V_y_/V_x_ (Fig. 5) but in practice it is difficult to reach a V_y_/V_x_ > 10, except for a small region at the top of the systems (between 0 and 0.1 in Fig. 5A). The effort needed to maintain a high V_y_/V_x_ throughout the system limits the possibilities to create small flow lanes in the entire system. Characterizing the influence of V_y_/V_x_ on the D_fc_/D_pore_ is essential to evaluate the performance of a specific device and/or operating conditions.

Overall, the results summarized in Fig. 5 show the correlation between the velocity of the fluid that flows in downstream direction (V_y_) and the fluid that flows into the pore (V_x_) with the size of the flow lanes. We expect that this balance also holds for conventional DLD devices. But unlike sparse or sieve-based systems, conventional DLD devices use geometric criteria to obtain stable (and periodic) V_y_/V_x_ to form flow lanes for displacing particles. Small changes in the geometry and/or inflow velocity of DLD devices can influence the stability and the periodicity of this balance, which may result in anisotropic permeability and different migration directions^20–24^.

## Conclusions

An in-depth characterization was done on the flow lane sizes in asymmetric, sieve-based deterministic lateral displacement (SLD) devices. CFD and μPIV were used to quantify the flow lanes, in addition to their flow velocities at different locations and at four different inlet velocities. The flow lanes width varies with the ratio of velocity components (V_y_/V_x_) and these velocity components (V_y_ and V_x_) vary depending on location and inlet velocity. The ratio of the longitudinal and transversal velocity components (V_y_ and V_x_) stabilized along the sieve towards the outlets. A good correlation was observed between the velocity ratio and the width of the flow lanes: a high V_y_/V_x_ ratio results in a smaller flow lane and vice versa. This implies that particles can also be displaced by accurate control of the hydrodynamics instead of only applying geometric design constraints. This insight may help application of this separation principle to larger-scale separation operation.

## Materials and Methods

### Experimental setup

**Figure 6:**
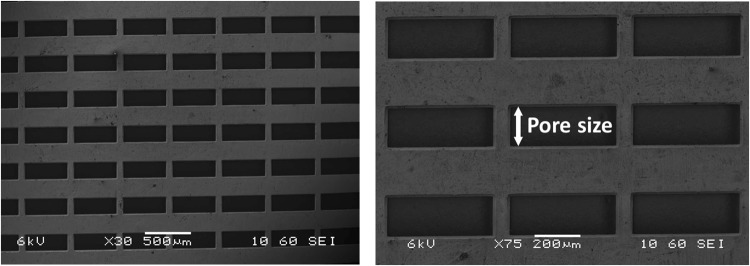
SEM images of a representative example of the microsieves used in our device with different magnification (x30 and x75). The pore size we discussed in this paper is indicated and is 200 ± 10 µm.

A sieve-based lateral displacement system was constructed with optical access from two sides (Fig. 3). The main frame was 3D printed from Polylactic acid (Ultimaker 2 + , The Netherlands) and two transparent Polymethylmethacrylate (PMMA) plates were attached to the front and at right side frame’s exterior. Inside, four nickel sieves (Veco, The Netherlands) were placed with 1 mm space between them at an average angle of 2.9° with respect to the channel walls (y-direction). The pores at the front of the sieves are 200 ± 10 µm × 500 ± 30 µm every 200 ± 10 µm in y-direction and every 50 ± 5 µm in z-direction (Fig. 6). Because of the electroforming production process, the pores at back of the sieves are slightly smaller than at the front. The channel length (y) was 100 mm, the width (x) 5 mm and the depth (z) 7 mm. The inlet was at the top and five outlets were at the bottom. The outlet tubes were used to fix specific outflow conditions of the five channels, outlets 1 to 4 were fixed at 16% of the inflow and outlet 5 at 36%^12^. The outlet suspension was collected in a collection vessel (1 L) and pumped (Masterflex L/S, Cole Parmer, US) to a pressure vessel (1 L) to dampen the pumping pulsations. The suspension was continuously recirculated through the system at the selected volumetric flow rate until the flow stabilized.

### Image recording

The flow was monitored with a camera at three positions in the system: the top section (0.1 normalized length) where the suspension enters, slightly above the middle section (0.4 normalized length) and the bottom section (0.8 normalized length) (Fig. 3). The motion of the fluid was visualized by seeding deionized water (milliQ) with 0.1 wt% red polystyrene tracer particles (d = 2 µm, ρ = 1.05 g/cm^3^, Microparticles GmbH) and 0.1 wt% non-ionic surfactant (Tween80) to prevent the particles from aggregating. The particles were illuminated with a thin (0.4 ± 0.1 mm) laser sheet (808 nm, Firefly, Oxford lasers) positioned in the middle of the channel at a depth of 3 mm. The reflected light was captured through a long distance magnifying lens (Navitar 1–14x) on a high speed camera (1024 × 1024 pixels, 20 × 20 µm^2^/pixel, Photron, SA1.1). The desired magnification (M), appropriate recording frequency and pulse length were chosen depending on the particle velocity. The recording frequency varied between 1–10 kHz and the pulse duration between 2–10 µs with a pulse power of 0.03–0.15 mJ/pulse. An overview of the full channel was taken with a magnification of M = 4, with the resolution of 1 pixel = 5 µm. Detailed images that focused on the pores of the largest sieve were taken at M = 10, with a resolution of 1 pixel = 2 µm.

### Flow lane determination using image analysis

The particle pathlines were visualized by superimposing 100 consecutively recorded images (Fig. 2). This new superimposed image only shows the maximum intensity of the all 100 images for each pixel position (z-stack, IMAGEJ, NIH). The flow lane width was defined as the distance between the sieve at the boundary transition of an obstacle and gap, and the most outward pathline that enters this gap (red line in Fig. 2). Using IMAGEJ, 45 flow lanes were measured in 15 superimposed images for each velocity at locations 0.4 and 0.8.

### Flow velocity calculation with Particle Image Velocimetry (PIV)

The image recordings were used to calculate full vector flow fields, which were calculated from the displacement of particles groups between two consecutive images by using commercial PIV software (DaVis). A multigrid cross correlation method was chosen with decreasing window size. The first interrogation window was 98 × 98 pixels, followed by a second calculation where the window size was 16 × 16 pixels when M = 4 and 32 × 32 pixels when M = 10. The flow fields derived from the recordings made of the whole channel (M = 4) had a higher resolution because the interrogation windows had a 50% overlap. The boundary of the vector field was defined using a geometrical mask to distinguish between regions were vector field should or should not be calculated. Following the vector field calculation, a post-processing algorithm was used to eliminate erroneous vectors and outlier detection was used based on the median value of the nearest neighbouring vectors^25^. If some vectors were rejected, they were interpolated or extrapolated from the accepted, neighbouring vectors. The percentage of rejected vectors from the obtained vector field was around 3 ± 2%. The final vector field was made by averaging the orthogonal components of nine consecutive vector fields (sliding averages).

### Experimental determination of V_y_ and V_x_

A line profile was made in the middle of the vector fields and in the middle the pores (e.g. the white line in Fig. 3). This line profile illustrates the average velocity in a volume of 0.08 mm thick when M = 4 and 0.06 mm when M = 10. The average velocity at the top (0.1) was calculated from 1500 PIV vector fields because the flow was turbulent. The flow at the middle and the bottom was observed to be stable (laminar) and the average velocity was determined from three sets of nine vector fields, each set was inconsistently taken at the beginning, in middle and at the end of the recording.

### 2D numerical simulations

Laminar fluid flow (NS equation) was numerically simulated in a 2D system with the same geometry as our experimental setup using COMSOL Multiphysics 5.3 (supplementary information). 2D simulations were used instead of 3D simulations to reduce computational resources and because in previous work minor differences were observed^12^. The fluid (water at 293.15 K) was assumed incompressible and the flow stationary. The flow was calculated for following average inlet velocities: 0.02 m/s (Re~100), 0.04 m/s (Re~200), 0.06 m/s (Re~300), 0.075 m/s (Re~375) and 0.12 m/s (Re~600). The outlets were fixed to specific outflow conditions, where outlets 1 to 4 were fixed at 16% of the inflow and outlet 5 at 36%^12^. The no-slip wall condition was applied and the mesh was refined until results were independent on the mesh (~200000 elements). The simulations were performed with the finite element method with 2^nd^ order elements for velocity and 1^st^ order elements for pressure. V_y_ and V_x_ were integrated over a cutline through each pore and the flow lanes were manually measured at the boundary transition of an obstacle and gap. Afterwards, three additional models were made with slightly varying geometry: two different angles (5.9° and 2.9°) and two different pore sizes (200 µm and 100 µm). All other conditions remained the same.

## Electronic supplementary material

Lateral displacement of a particle

A particle passing the microsieve

Velocity and Pressure distribution in a sieve-based lateral displacement (SLD) system

## Data Availability

The datasets generated during and/or analysed during the current study are available from the corresponding author on reasonable request.
