# Supplementary material for: Visualizing the hydrodynamics in sieve-based lateral displacement systems
Source: Sci Rep. 2018 Aug 27;8:12861. doi: 10.1038/s41598-018-31104-2 (PMC6110767; doi:10.1038/s41598-018-31104-2)
Supplement: Supplementary file 3 — Velocity and Pressure distribution in a sieve-based lateral displacement (SLD) system [file 41598_2018_31104_MOESM3_ESM.pdf]

Supplementary information for:

Visualizing the hydrodynamics in sieve-based lateral displacement systems

J.P. Dijkshoorn[ab], J.C. de Valença[b], R.M. Wagterveld[b], R.M. Boom[a], M.A.I. Schutyser[a*]

[a] Laboratory of Food Process Engineering, Wageningen University, Bornse Weilanden 9, 6708WG, Wageningen, The Netherlands
[b] Wetsus, European Centre of Excellence for Sustainable Water Technology, Oostergoweg 9, 8911MA, Leeuwarden, The Netherlands

*Velocity distribution in the whole system (m/s)

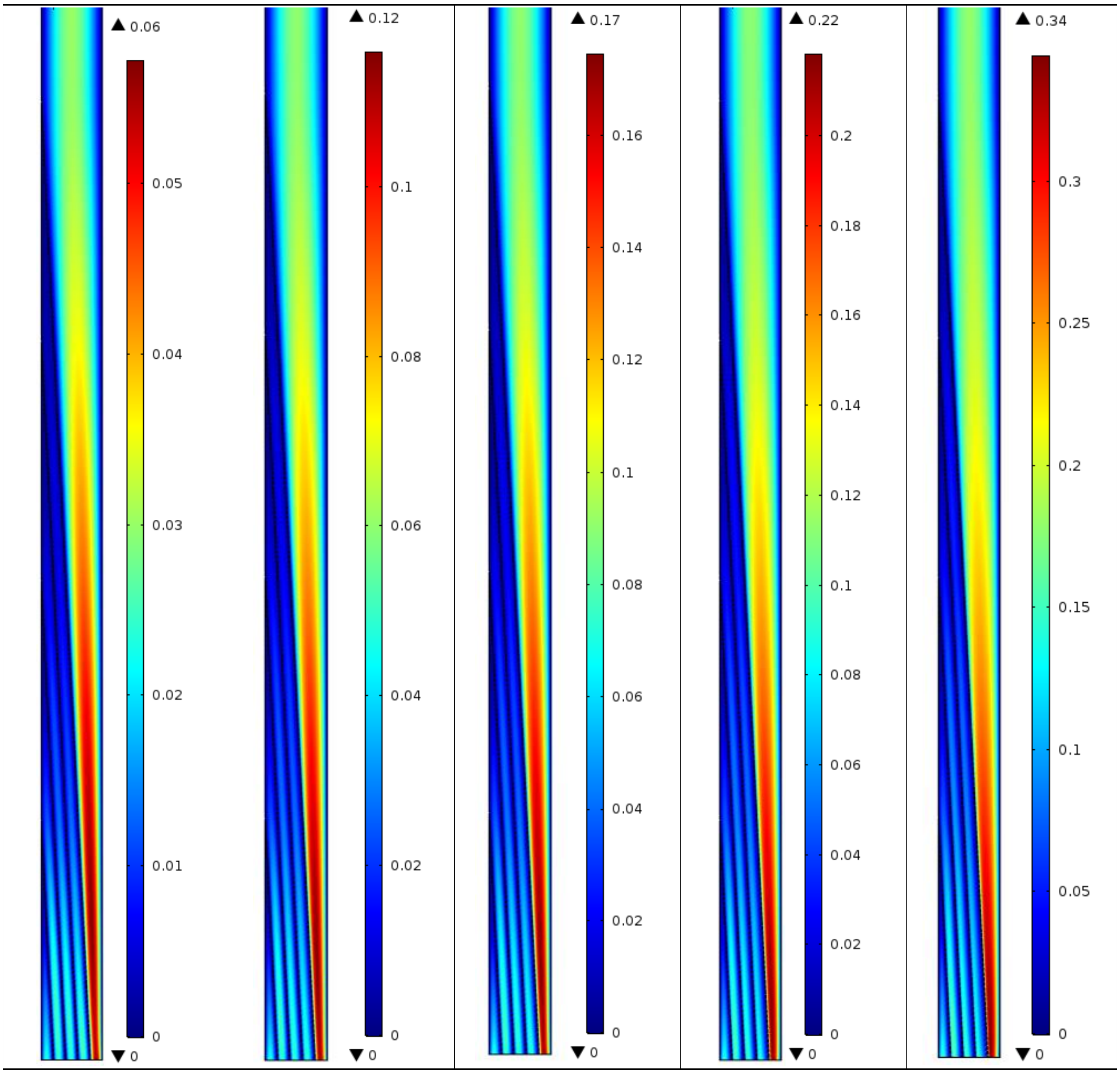

0.02 m/s	0.04 m/s	0.06 m/s	0.075 m/s	0.12 m/s

Figure.1: Velocity distribution in the whole system for an average inlet velocity of 0.02 m/s, 0.04 m/s, 0.06 m/s, 0.075 m/s and 0.12 m/s respectively. Inlet at the top and the five outlets are at the bottom, four sieves are placed inside at an angle of 2.9° (as mentioned in the Materials and Methods).

Pressure distribution in the whole system (Pa)

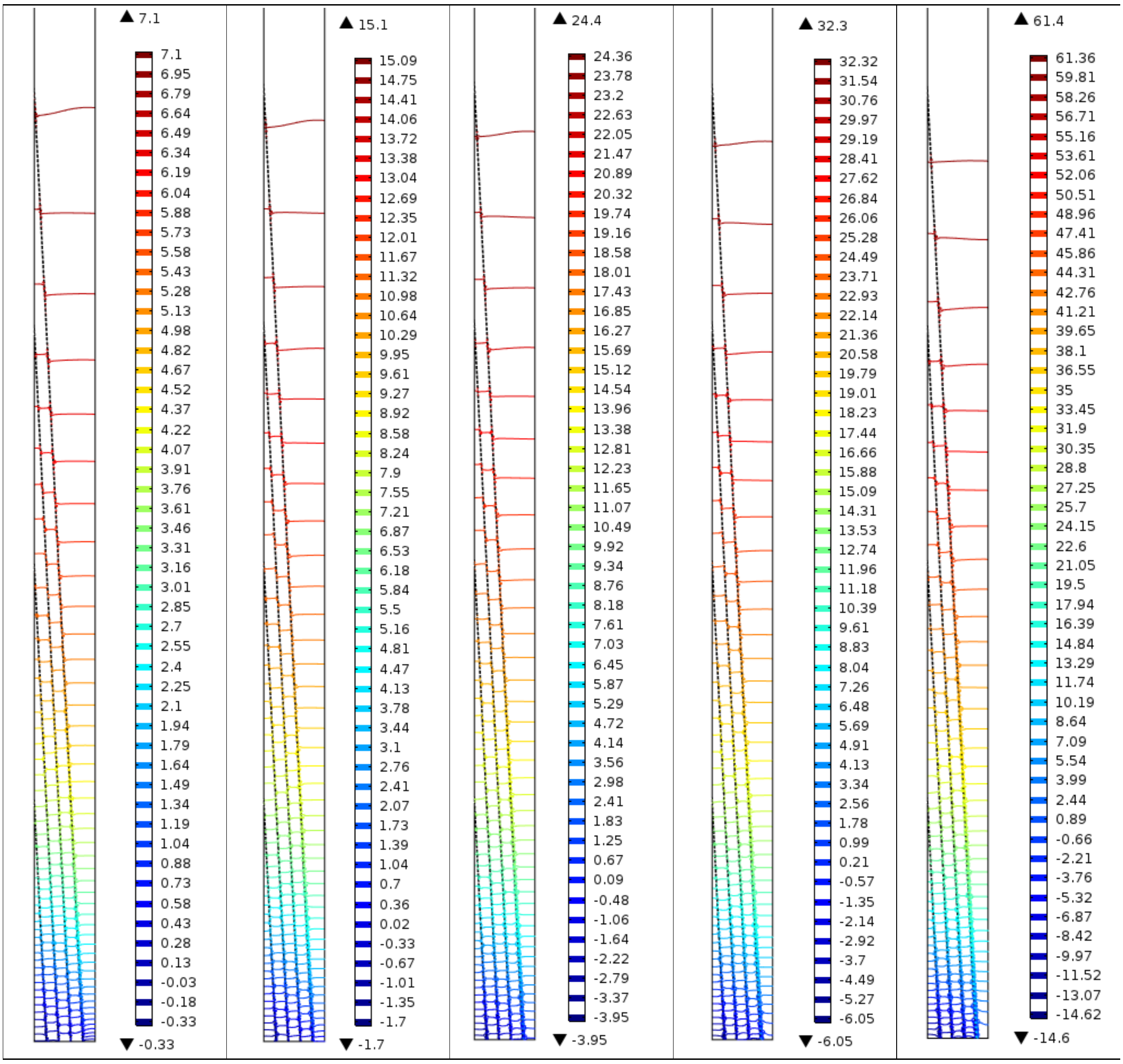

0.02 m/s	0.04 m/s	0.06 m/s	0.075 m/s	0.12 m/s

Figure 2: Pressure distribution (Pa) in the whole system for an average inlet velocity of 0.02 m/s, 0.04 m/s, 0.06 m/s, 0.075 m/s and 0.12 m/s respectively. The pressure becomes negative because only the right-most outlet has pressure-based boundary conditions, the other outlets are fixed (as mentioned in the Materials and Methods).
